# Domestication and Improvement in the Model C4 Grass, Setaria

**DOI:** 10.3389/fpls.2018.00719

**Published:** 2018-05-29

**Authors:** Hao Hu, Margarita Mauro-Herrera, Andrew N. Doust

**Affiliations:** Department of Plant Biology, Ecology, and Evolution, Oklahoma State University, Stillwater, OK, United States

**Keywords:** Setaria, foxtail millet, genetic control, domestication, shattering, plant architecture, flowering time, photoperiod

## Abstract

*Setaria viridis* (green foxtail) and its domesticated relative *S. italica* (foxtail millet) are diploid C4 panicoid grasses that are being developed as model systems for studying grass genomics, genetics, development, and evolution. According to archeological evidence, foxtail millet was domesticated from green foxtail approximately 9,000 to 6,000 YBP in China. Under long-term human selection, domesticated foxtail millet developed many traits adapted to human cultivation and agricultural production. In comparison with its wild ancestor, foxtail millet has fewer vegetative branches, reduced grain shattering, delayed flowering time and less photoperiod sensitivity. Foxtail millet is the only present-day crop in the genus *Setaria*, although archeological records suggest that other species were domesticated and later abandoned in the last 10,000 years. We present an overview of domestication in foxtail millet, by reviewing recent studies on the genetic regulation of several domesticated traits in foxtail millet and discuss how the foxtail millet and green foxtail system could be further developed to both better understand its domestication history, and to provide more tools for future breeding efforts.

## Introduction

The evolution of human civilization is in many ways written on the back of grass domestication. Cereal grains directly or indirectly provide approximately half of the calories that support human populations (Emily et al., 2013). Furthermore, forage provided by both wild and improved grasses feed domesticated animal herds in much of the world. Grasses have also figured prominently in unraveling the domestication process and its relationship to evolution via natural selection (Doust, 2007a; Glemin and Bataillon, 2009). Despite this importance, differences in the details of domestication amongst the various lineages of grasses raise the question of whether there is a single model of domestication in the grasses or unique paths to similar phenotypes. Some phenotypes appear conserved (at least for cereal crops like rice, wheat, and maize), such as the change from a freely dispersed propagule to one that is hard to dislodge, and therefore reliably harvested from the plant (“non-shattering") (Purugganan and Fuller, 2009). However, even this change, which has the practical result of retaining seeds on the plant, appears to involve different combinations of floral and inflorescence parts, and be controlled by different genes in different domestication events (Doust et al., 2014b). In this context, it is worth considering whether increasing the number of cereal domestication events that we study can help us better understand underlying processes of domestication. Here, we use the term ‘event’ to signify an independent domestication, while recognizing that the process of domestication is not an instantaneous change from wild to domesticated forms (Allaby et al., 2017). In recent years, insights into the domestication process have increased rapidly, in part driven by the increased ease of genome sequencing. Rice (*Oryza sativa*)was the first sequenced grass genome (Goff et al., 2002; Yu et al., 2002; Matsumoto et al., 2005), followed by sorghum (*Sorghum bicolor*) (Paterson et al., 2009), *Brachypodium* (Vogel et al., 2010), maize (*Zea mays*) (Schnable et al., 2009), and various others, including foxtail millet (*Setaria italica*) (Bennetzen et al., 2012; Zhang et al., 2012), pearl millet (*Pennisetum glaucum*) (Varshney et al., 2017), *Oropetium* (VanBuren et al., 2015), and *Dichanthelium* (Studer et al., 2016). A new wave of investigation has recently started to target the wild progenitors of crop species, including the ancestor of maize, teosinte, and several ancestors of wheat (Jia et al., 2013a; Ling et al., 2013; Yang et al., 2017). These resources are providing new insights into domestication, and new opportunities for selection and improvement of under-utilized grain crops.

**FIGURE 1:**
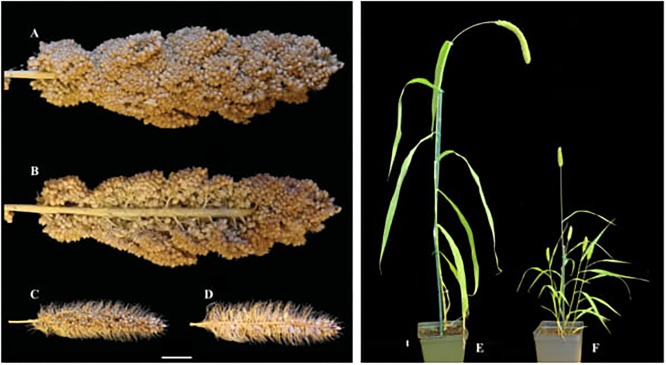
Morphological comparison of domesticated foxtail millet and its wild ancestor green foxtail. Whole and longitudinal section of foxtail millet **(A,B)** and green foxtail **(C,D)** panicles. Whole plant of foxtail millet **(E)** and green foxtail **(F)**.

Foxtail millet (*S. italica*) is an ancient cereal grain crop of Northern China, that was grown in rotation with winter wheat in the regions around the Yellow River (Diao and Jia, 2017b). It is now grown in China, India, and Africa, excelling as a drought and low nutrient tolerant grain (Goron and Raizada, 2015; Nadeem et al., 2018). Its wild ancestor, green foxtail (*S. viridis*), is a widespread weed of temperate regions worldwide (Dekker, 2003). Foxtail millet principally differs from green foxtail in reduced vegetative branching, greater height, increased inflorescence branching, and larger seeds (**Figure 1**). Draft genomes are now available for both foxtail millet (*S. italica*) and its wild ancestor, green foxtail (*S. viridis*) (*Setaria viridis* v1.1, DOE-JGI^1^).

The genus *Setaria* belongs to the subtribe Cenchrinae and tribe Paniceae within the subfamily Panicoideae (Kellogg, 2017). All species possess the C4 photosynthetic pathway, and close relatives include pearl millet (formerly *Pennisetum glaucum*, now *Cenchrus glaucum*), (Chemisquy et al., 2010), napier grass (*C. purpureum*), and switchgrass (*Panicum virgatum*). The sister tribe is the Andropogoneae, which includes the important crop species maize (*Z. mays*), sorghum (*Sorghum bicolor*), and sugar cane (*Saccharum officinarum*) (Doust et al., 2009). *Setaria* is the largest genus in the Cenchrinae, and consists of approximately 100 species that are widely distributed in warm and temperate regions worldwide (Kellogg, 2017). The known domesticated crops in this genus include foxtail millet, yellow foxtail (*S. pumila*), and plains bristle grass (*S. macrostachya*), but foxtail millet is the only one that remains a major crop (Austin, 2006; Diao and Jia, 2017b). Other members of the genus, including green foxtail, are invasive weeds in corn and soybean field in many parts of the world (Dekker, 2003; Darmency et al., 2017). Besides *Setaria* and *Cenchrus*, the Cenchrinae clade also includes several smaller genera whose evolutionary relationships are not well understood (Doust et al., 2007; Kellogg, 2015, 2017). The relationships of foxtail millet and its wild progenitor, green foxtail, with respect to other species in the genus are unclear, as all studies to date have shown *Setaria* to be either para- or polyphyletic, depending on its circumscription (Doust et al., 2007; Kellogg et al., 2009; Kellogg, 2015). However, foxtail millet and green foxtail belong to a small well-supported clade, along with the tetraploid species, *S. verticillata* and *S. faberi* (Wang et al., 2009). *S. faberi* is an allotetraploid, with both genomes closely related to green foxtail, whereas *S. verticillata* appears to have one genome related to green foxtail, and the other to more distantly related species (Kellogg et al., 2009; Layton and Kellogg, 2014; Kellogg, 2017). The clade appears to be Asian in origin, even though the three wild species are all now widely distributed weeds.

Genetic analyses on foxtail millet, which date from at least the 1930’s, concentrated on the genetics of distinct phenotypes, such as spikelet-tipped bristles and purple bristles (Ayyangar et al., 1933). Experiments on crossing in foxtail millet were also performed, and the difficulties in crossing the small flowers of these inbreeding plants was first discussed (Li et al., 1935). Much breeding work continued in China over the years since that time (Diao and Jia, 2017a), but only a few population genetic and herbicide resistance papers were published in English until the end of the last century. These included investigations into the origin of domestication of foxtail millet, which used a variety of markers to test hypotheses of origins in Northern China, South Asia, and the hypothesis of twin origins, in both China and Europe (Kawase and Sakamoto, 1984; Jusuf and Pernes, 1985; Darmency et al., 1987; Wang et al., 1995b; Fukunaga et al., 1997; Li et al., 1998; Nakayama et al., 1998; Le Thierry d’Ennequin et al., 2000). More recently, comparative genomic studies used common SSR markers to infer syntenic relationships between multiple grass genomes, including foxtail millet (Wang et al., 1998), and showed how the nine chromosomes of *Setaria* are simple rearrangements of the twelve chromosomes of rice (Brutnell et al., 2015). In addition, the same mapping population used to create the genetic maps that were the basis of comparative genome analyses was later used to identify quantitative trait loci (QTL) underlying phenotypic traits of both the vegetative body and inflorescence of *Setaria* (Doust et al., 2004, 2005; Doust and Kellogg, 2006). After the creation of advanced generation RILs, this population was also used to analyze the genetic determinants of traits such as branching (Mauro-Herrera and Doust, 2016; Doust, 2017), shattering (Doust et al., 2014a; Odonkor, 2015), height (Mauro-Herrera and Doust, 2016; Doust, 2017; Feldman et al., 2017), flowering time (Mauro-Herrera et al., 2013; Doust, 2017), and biomass (Mauro-Herrera et al., 2013; Mauro-Herrera and Doust, 2016; Doust, 2017; Feldman et al., 2017; Banan et al., 2018).

In 2012, the Setaria reference genome was independently released by two groups from the United States (Bennetzen et al., 2012) and China (Zhang et al., 2012). The United States group sequenced the foxtail millet inbred line *Yugu1* using the Sanger platform, and generated a 396.7 Mb assembly with 80% genome coverage and 35,158 annotated loci (Bennetzen et al., 2012). The Chinese group reported a genome from cultivated foxtail millet cv. *Zhanggu* sequenced with the Illumina platform, producing a ∼423 Mb draft that covered ∼86% of the total genome, with 38,801 genes annotated (Zhang et al., 2012). The genome assemblies allowed many more markers to be identified and denser linkage maps to be created, resulting in increased precision of QTL analysis (Kumari et al., 2013; Mauro-Herrera et al., 2013; Pandey et al., 2013; Zhang et al., 2014; Fang et al., 2016; Mauro-Herrera and Doust, 2016). Further reduced representation sequencing has also allowed dense marker maps to be generated for mapping populations (Fahlgren et al., 2015; Feldman et al., 2017). The genome sequence of the wild parent, green foxtail, of the Wang et al. (1998) mapping population is available (acc. *A10.1*), although there is as yet no publicly available genome for the foxtail millet accession, *B100*, the female parent of this population. Several other mapping populations have been created, both between foxtail millet and green foxtail (Qie et al., 2014), and within foxtail millet (Zhang K. et al., 2017), although none of them have been used to directly address the genetic changes that occurred during domestication.

In the last 10 years, the Setaria system (comprising foxtail millet and its wild progenitor green foxtail) has been promoted as an experimental model system for studying grass genetics and biofuel production (Doust et al., 2009; Brutnell et al., 2010, 2015; Li and Brutnell, 2011) (**Figure 1**). Both species possess several significant advantages over related C4 panicoid grasses such as maize and sorghum, including short life cycle, small size, simple growth requirements and ease of transformation (Brutnell et al., 2010). Difficulties with making controlled crosses between accessions, owing to small size of the individual flowers, has been addressed successfully by both temperature and chemical interventions to kill pollen in the female parent (Jiang et al., 2013; Zhang H.Y. et al., 2017), and much work has also been done on overcoming seed dormancy issues (Sebastian et al., 2014; Brenner et al., 2015; Acharya et al., 2017). Following recommendations made at the First international Setaria Genetics Conference in Beijing in 2014, we have adopted the name Setaria (capitalized, no italics) for this model system (foxtail millet and green foxtail) while *Setaria* (capitalized, italics) remains the correct form for the genus name (Diao et al., 2014; Doust and Diao, 2017).

## The Domestication of Foxtail Millet

The most recent evidence to date suggests that domestication of foxtail millet occurred between 9,000 and 6,000 YBP in China, before being brought west into Europe (Diao and Jia, 2017b). Other hypotheses have included origins in Europe and in west Asia (Jones, 2004; Nasu et al., 2007). A separate origin in Europe is based on the relatively early appearance of foxtail millet grains in the European archeological record (4,000–3,000 YBP) (Nasu et al., 2007), and genetic evidence that European foxtail millet genotypes are more closely related to co-occurring green foxtail genotypes than to foxtail millet from China (Le Thierry d’Ennequin et al., 2000). However, recent studies of the movement of starchy grains across Eurasia have concluded that movement can be much faster by trade than formerly assumed (Lightfoot et al., 2013), and that the close relationship of foxtail millet and green foxtail in Europe is likely the result of hybridization between foxtail millet and green foxtail in Europe (Le Thierry d’Ennequin et al., 2000; Austin, 2006). Beyond archeological evidence, a recent genomic study that sequenced 916 foxtail millet accessions suggested a single domestication event of foxtail millet in China (Jia et al., 2013b). Green foxtail accessions in China show a strong relationship to Chinese foxtail millet accessions, indicative of continued gene flow after domestication. The Chinese green foxtail accessions are distinct from populations in North America, some of which apparently arrived there prior to European settlement (Huang et al., 2014; Huang and Feldman, 2017; Schroder et al., 2017).

A recent review of archeological finds from China suggests that the domestication and widespread adoption of foxtail millet in China involved three phases (Diao and Jia, 2017b). The first was the pre-domestication phase (23,000 to 9,000 YBP), where several archeological finds of plant starches and stone tools for processing green foxtail and/or foxtail millet seeds have been found (Yang et al., 2012; Jia et al., 2013b). However, intact grains have not been recovered for this phase. The second phase was that of domestication (9,000 to 6,000 YBP), where foxtail millet grains have been found in multiple archeological sites in northern China, especially in the Yellow River region (Lü et al., 2009). These finds indicate that foxtail millet was being cultivated as a food crop. The third phase was the expansion phase (after 6,000 YBP), a phase during which large quantities of foxtail millet grains have been found in hundreds of archeological sites, especially in southern and western China (Lü et al., 2005, 2014). The evidence from archeological sites in the expansion phase suggests that foxtail millet had spread to several major regions of ancient China, and that cultivation had become widespread. In addition, after 6,000 YBP, foxtail millet had quickly spread to other regions, and foxtail millet grains have been recovered from archeological sites from Eastern Siberia (5,550–4,050 YBP) (Sergusheva and Vostretsov, 2009), Korea (5,500 YBP) (Lee, 2011), and Japan (4,000 YBP) (Crawford, 1992).

## The Genetics of Domestication

Although green foxtail and foxtail millet are usually described as separate species (Austin, 2006), recent studies have increasingly led to the conclusion that foxtail millet is just a domesticated version of green foxtail (Doust et al., 2009). The genetic evidence includes both isozyme (Wang et al., 1995a) and DNA marker analyses (Wang et al., 1998; Hirano et al., 2011; Li et al., 2012). Wang et al. (2010), using 50 accessions of cultivated foxtail and 34 of green foxtail, determined that foxtail millet only contained 55% of the diversity of green foxtail, and that domestication occurred in China approximately 8700 years ago. They did not find fixed differences between the cultivars and their wild relatives, but did find a high proportion of shared polymorphisms, particularly for the cultivars. On average, domesticated foxtail millet shared almost 75% of its polymorphisms with green foxtail, whereas the proportion for green foxtail was 36%. Unique polymorphisms were present in both species, but were much less prevalent in cultivars than in the wild species. They suggested that the domestication bottleneck in foxtail millet was more severe than in maize, but slightly less than in rice. More recent studies, on Chinese and on two sets of North American collections, have provided new insights into the relationships and diversity of foxtail and green foxtail. The large degree of overlap found by Wang et al. (2010) in Chinese foxtail millet and green foxtail accessions may be due to their long shared history, as recent analyses of green foxtail accessions from North America, China, and other regions, found two main groups in North America, and a third group that was more foxtail millet-like, and to which all of the Chinese accessions belonged (Huang et al., 2014; Schroder et al., 2017). A recent sequence study of multiple varieties of foxtail millet identified selective sweeps between landrace and improved varieties indicative of selection after domestication, as well as regions of low diversity in all varieties, indicative of a domestication bottleneck (Jia et al., 2013c). Jia et al. (2013c) focused on a low-diversity region on chromosome 9 that contains the SH1 locus, whose orthologous locus in sorghum has been shown to control abscission of the grain (shattering) (Lin et al., 2012). Further analysis of the genetic signature of domestication is necessary to understand the extent to which domestication in foxtail millet is similar to processes in other cereal grains.

## Genes Contributing to Domestication-Related Traits

The identification of genes responsible for domestication traits in Setaria is still in its early stages, although multiple studies have highlighted genomic regions which appear to harbor causal loci (Doust et al., 2004, 2005, 2017; Mauro-Herrera et al., 2013; Odonkor, 2015; Mauro-Herrera and Doust, 2016; Coelho et al., 2017; Feldman et al., 2017; Wang et al., 2017). Some of these also appear to colocate with regions controlling domestication or improvement traits in other species (Mauro-Herrera et al., 2013; Doust et al., 2014b). Conservation of genes responsible for domestication was first noted by Paterson et al. (1995), and may indicate a shared genetic mechanism for changes in traits between wild and domesticated species. However, Bennetzen et al. (2012) compared sequences of several candidate domestication genes, such as *Q*, *qSH1*, *tb1*, etc., between foxtail millet and green foxtail, and found no coding differences, suggesting that the domestication of foxtail millet involved either a different set of loci, or regulation mechanisms that were not obvious by simple sequence comparison. Further work has refined and extended these observations, and we discuss below the evidence for genetic control of several domestication and improvement phenotypes.

### Shattering

Seed shattering, or seed dispersal, is the mechanism by which plants disperse seeds at maturity. However, it is an unfavorable trait for cereal crops in agricultural production, because it leads to reduction in harvesting and grain yield (Li and Olsen, 2016). The loss of the seed-shattering habit is thought to be one of the most important events in seed crop domestication, and it is a critical feature that distinguishes modern crops from their wild progenitors (Li and Olsen, 2016). In grasses, the position and method of seed shattering is regulated by where abscission zones are formed. An abscission zone (AZ) can be formed in a variety of positions on the pedicel of the spikelet or on subtending branches, but in Setaria is underneath the glumes, so that the whole spikelet, containing one seed, falls off as a unit (Doust et al., 2014b). Hodge and Kellogg (2016) studied the anatomy and histology of AZs in both green foxtail and foxtail millet, but, unlike sorghum, rice or barley, no notable lignification was observed in the AZ of Setaria. However, cellular staining identified subtle differences of cell size, orientation and arrangement between green foxtail and foxtail millet, particularly in the non-shattering foxtail millet line *Yugu1*. The tensile strength that is required to remove a spikelet from the pedicel is much lower in wild green foxtail than domesticated foxtail millet, and a discrete cup-shaped structure remains after dispersal of the grain. In foxtail millet, however, a force able to remove the grain from the inflorescence usually results in a break in the pedicel rather than in the abscission zone beneath the glumes (Doust et al., 2014a).

**FIGURE 2:**
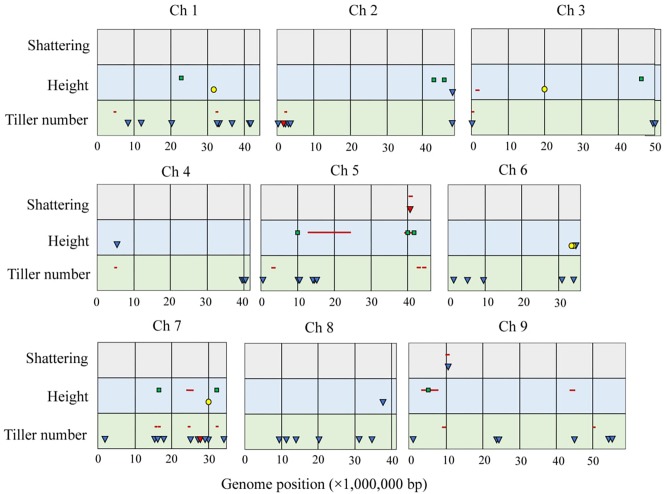
Summary of domesticated and modern improvement loci across the Setaria genome. Each box represents an individual chromosome, where the values along the *x*-axis are physical position in the foxtail millet genome, *y*-axis indicates different traits. In each trait, the first row represents the foxtail millet × green foxtail F7 RIL population used in studies of Doust et al. (2014a) and Mauro-Herrera and Doust (2016) (QTLs displayed by red bars), and Feldman et al. (2017) (QTLs indicated by green squares). The second row represents the foxtail millet population studies of Jia et al. (2013b) (blue triangles correspond to GWAS signals of modern improvement, and red triangles show selective sweeps), and Fang et al. (2016) (QTLs represented by yellow circles).

Doust et al. (2014a) evaluated seed shattering in an F7 RIL mapping population of Setaria, and identified two significant QTLs – one locus (QTL1) on chromosome 9 that contributes more than 35% of the phenotypic variation, and the other locus (QTL2) on chromosome 5 that is responsible for about 8% (**Table 1**). They also found that there was no significant genetic interaction between QTL1 and any other loci, including QTL2, whereas QTL2 shows epistatic interactions with several other genomic locations. QTL1 is syntenic to the region on sorghum chromosome 1 that contains the SH1 locus, identified as the major locus controlling shattering in that species (Lin et al., 2012). The same locus was also detected in an analysis of genome variability in 916 diverse foxtail millet varieties (Jia et al., 2013b), where an 855-bp indel in coding sequence was found when compared with green foxtail (**Figure 2**). In addition, all foxtail millet varieties show very low sequence diversity in this region when compared with the wild progenitor green foxtail. Odonkor (2015) identified the sorghum SH1 homologous in chromosome 9 as a candidate gene for seed shattering in foxtail millet, and suggested that a miniature inverted-repeat transposable element (MITE) insertion leads to the non-shattering phenotype in foxtail millet. These lines of evidence strongly suggest that the *Setaria* ortholog of the SH1 locus controls much of the shattering phenotype in and Setaria. The second QTL, QTL2, identified by Doust et al. (2014a), is overlapped by a region showing a strong selective sweep in the GWAS study by Jia et al. (2013b) (**Figure 2**). However, the selective sweep differentiates landrace and modern varieties, with greater diversity in landrace varieties, suggesting that QTL2 was selected upon after domestication and during the improvement of the crop. This region harbors the ortholog of *qSH1*, a major seed shattering gene in rice. If the *qSH1* homolog is confirmed as the causal gene in QTL2 it may suggest that it was selected upon during crop improvement rather than in the initial stages of domestication. Thus for shattering it is possible that there is a shared conserved genetic mechanism that spans multiple species.

### Plant Height

Height is a trait that may be targeted by human selection during crop domestication and improvement, and there is significant variation in height in landrace varieties of foxtail millet, and within green foxtail accessions (Li and Yang, 2008; Chander et al., 2017a,b; Ghimire et al., 2018). Differences in height amongst landrace varieties may reflect both an ancestral increase in height took place as selection for losing unproductive tillers, followed by differentiation as farmers in different localities targeted height for a variety of reasons, including grain production and foraging. The GWAS analysis of 916 foxtail millet varieties by Jia et al. (2013b) documented genomic regions showing strong association with plant height on chromosomes 2, 4, and 6, while Fang et al. (2016) used an F2 population from a cross between two foxtail millet varieties, and found four height related QTLs on chromosomes 1, 3, 6, and 7. Recent studies on height in the F7 recombinant inbred line mapping population derived from that of Wang et al. (1998) identified major height-related QTLs on chromosomes 5, 7, and 9, with the effect of the different QTL being expressed at different developmental stages. Thus, for example, the QTL on chromosome 5 controls at least half of the variation in height at the seedling stage, but less variation at flowering and during seed maturation (Mauro-Herrera and Doust, 2016; Feldman et al., 2017). Only a few QTL overlap between the different studies (**Figure 2**), suggesting that both environmental and genotypic variation between trials contribute to differences in genetic regulation. The genes underlying these QTL have yet to be definitively isolated, but mapping efforts of several mutants are ongoing (Xue et al., 2016; Fan et al., 2017). The QTL regions also contain known candidate genes for height change, including, on chromosome V, the ortholog of *SEMIDWARF 1* (*SD1*), a gene that was important in breeding semi-dwarf rice varieties for the Green Revolution (Ashikari et al., 2002; Asano et al., 2011). Further work needs to be done to show that these genes actually control height, or to explain how different genes control height at different developmental stages.

### Plant Branching

Vegetative branching significantly contributes to the determination of plant architecture (Doust, 2007a,b; McSteen, 2009; Hodge and Doust, 2017), and involves complicated genetic and environmental regulation (Finlayson et al., 2010). Human-directed selection and domestication has led to a dramatic reduction in vegetative branching in Setaria, presumably as a response to increased planting density and increases in seed weight and number of seeds per inflorescence (Doust, 2007a). Doust et al. (2004) reported the first QTL analysis of basal (tillering) and aerial branching in Setaria, and identified four QTLs for tillering on chromosomes 1, 3, and 4, and four QTLs for aerial branching on chromosomes 4, 5, and 9. Interestingly, the ortholog of *tb1*, a major gene controlling branching in maize, was found to associate with only a minor QTL for branching in Setaria (Doust et al., 2004), as was also reported in the closely related species, pearl millet (Poncet et al., 2002). A further study of trials in different greenhouse conditions and between greenhouse and field trials revealed substantial genotype by environment (G × E) relationships (Doust and Kellogg, 2006; Mauro-Herrera and Doust, 2016). Comparison with the GWAS analysis of 916 foxtail millet varieties by Jia et al. (2013b) showed several regions of overlap but also many unique QTL, emphasizing that branching has a high G × E component (**Figure 2**).

The size and branching architecture of the inflorescence (panicle) also directly affects yield, as it determines the number of grains per panicle and hence final grain yield. Foxtail millet and green foxtail differ in the number of orders of branching within the inflorescence (**Figures 1A–D**), and Doust et al. (2005) identified three QTL for primary branch number/density on chromosomes 3, 7, and 9, whilst Jia et al. (2013b) identified three GWAS associations of chromosomes 2 and 5 (**Table 1**). Several of the QTL identified for primary inflorescence branch number in Doust et al. (2005) overlapped with QTL identified for vegetative branching (Doust et al., 2004). Other studies have focused on traits indicative of total yield, including panicle length, diameter, and weight (Jia et al., 2013b; Wang et al., 2017; Zhang K. et al., 2017). However, few of these QTL overlap, suggesting multiple genes underlying these traits and/or environmental regulation. However, the latter seems surprising given the tight control over inflorescence form exhibited by plants, and it may be that present methods of analysis have not been able to satisfactorily decompose the inflorescence branch system in a way that can reveal underlying developmental drivers. An attempt to do this was done within the clade that *Setaria* belongs to (Doust and Kellogg, 2002), but a detailed analysis of inflorescence development in Setaria using emerging technologies such as x-ray tomography and other non-invasive techniques, along with new ways of analyzing data such as persistent homology analysis, remains to be accomplished (Bucksch et al., 2017).

**Table 1:** Summary of published quantitative trait loci (QTLs) of key domestication traits in Setaria.

Trait	Method	Population	Chromosome and candidate genes	Reference
Shattering	QTL	*S. viridis* ×*S. italica*	5: Seita.5G381300 (*qSH1*)	Doust et al., 2014a
			9: Seita.9G154300 (*SbSH1*)	
	QTL	*S. viridis* ×*S. italica*	9: Seita.9G154300 (*SbSH1*)	Odonkor, 2015
	Comparative mapping	*S. italica*	9: Seita.9G154300 (*SbSH1*)	Lin et al., 2012
	GWAS	*S. italica*	9: Seita.9G154300 (*SbSH1*)	Jia et al., 2013b
Branching	QTL	*S. viridis × S. italica*	1: Seita.1G136300 (*AtSUR1*)	Doust et al., 2004;
				Mauro-Herrera and Doust, 2016
			3: Unknown	
			4: Seita.4G219200 (*OsMOC1*)	
			5: Unknown	
			6: Unknown	
			9: Seita.9G034300 (*AtAXR1*), Seita.9G123400 (*ZmTB1*)	
	QTL	*S. viridis × S. italica*	5: Seita.5G368600 (*ZmBA1*)	Doust and Kellogg, 2006
			9: Seita.9G123400 (*ZmTB1*)	
	GWAS	*S. italica*	1: Unknown	Jia et al., 2013b
			2: Unknown	
			5: Unknown	
			8: Unknown	
			9: Unknown	
	QTL	*S. italica*	1: Unknown	Fang et al., 2016
			7: Unknown	
Height	QTL	*S. viridis × S. italica*	5: Seita.5G404900 (*OsSD1*)	Mauro-Herrera and Doust, 2016
			9: Unknown	
	QTL	*S. viridis × S. italica*	2: Seita.2G291300 (*ZmDFL1*)	Feldman et al., 2017
			5: Seita.5G404900 (*OsSD1*)	
			9: Unknown	
	GWAS	*S. italica*	2: Unknown	Jia et al., 2013b
			4: Unknown	
			6: Unknown	
	QTL	*S. italica*	1: Unknown	Fang et al., 2016
			3: Unknown	
			6: Unknown	
			7: Unknown	
	QTL	*S. italica*	1: Unknown	Wang et al., 2017
Inflorescence branching	QTL	*S. viridis* ×*S. italica*	3: Unknown	Doust et al., 2005
			7: Seita.7G222300 (*ZmFL1*)	
			9: Unknown	
	GWAS	*S. italica*	2: Unknown	Jia et al., 2013b
			5: Unknown	
Flowering time	QTL	*S. viridis* ×*S. italica*	4: Seita.4G067600 (*OsHD3a*)	Mauro-Herrera et al., 2013
			8: Seita.8G034000 (*ZmTFL1*)	
Flowering time	QTL	*S. viridis* ×*S. italica*	2: Seita.2G436800 (*AtCDF1*),	Doust, 2017; Doust et al., 2017
			Seita.2G444300 (*SbPRR37*)	
			3: Seita.3G307200 (*OsREF6*)	
			4: Seita.4G116600 (*OsHD1*/*CONSTANS*)	
			5: Seita.5G317600 (*ZCN12*)	
			8: Seita.8G040100 (*OsPRR59*)	
	GWAS	*S. italica*	1: Seita.1G236100 (*OsTOC1*), Seita.1G304900 (*OsHd1*), Seita.1G334800 (*OsCOP1*)	Jia et al., 2013b
			2: Seita.2G436800 (*OsCDF3*), Seita.2G444300 (*OsPPR37*)	
			3: Seita.3G044600 (*OsTOE1*)	
			4: Seita.4G282900 (*OsFD1*)	
			6: Seita.6G240100 (*OsABF3*), Seita.6G248900 (*OsSPY*)	
			7: Seita.7G007800 (*OsP*), Seita.7G119400 (*OsCRY1b*)	
			8: Seita.8G146800 (*OsGF14d*), Seita.8G146900 (*OsFKF1*)	
			9: Seita.9G342700 (*OsMADS56*)	

### Flowering Time and Photoperiod Sensitivity

Flowering is a critical indicator of the transition from vegetative growth to reproduction in the development of plants. The wild progenitors of crop species typically are photoperiod sensitive, because they are required to maximize their adaptability to their growing location. Crop species, on the other hand, may be selected for reduced photoperiod sensitivity in order to increase the range of localities in which they can be grown, and may have longer growing time, later flowering, and thus increased yield (Cockram et al., 2007). Thus selection on flowering time is unlikely to be a direct response to domestication but is best considered as a target for crop improvement. However, flowering time and photoperiod sensitivity also interact with other important traits, such as branching, height and biomass (Doust, 2017;Doust et al., 2017), making it relevant to discuss here.

Setaria has a wide latitudinal and longitudinal distribution around the world, and genetic evidence suggests that foxtail millet was domesticated from green foxtail in northern China, at temperate latitudes. Both foxtail millet and green foxtail show variation in photoperiod sensitivity (Swanton et al., 1999; Li and Yang, 2008; Jia et al., 2013b). In China, the four different growing regions are distinguished on the basis of spring or summer growing season and on variation in sensitivity to photoperiod and altitude (Diao and Jia, 2017a). Jia et al. (2013b) grew 916 accessions in four different latitudinal localities, and recorded differences in both morphology and heading date, along with differing significant SNP associations. A recent multiplexed shotgun genotyping (MSG) resequencing analysis of 439 RILs (derived from a cross between foxtail millet accessions Zhanggu and A2) under short (<12 h light) and long photoperiods (>14 h light), uncovered a total of 59 QTLs for 14 agronomic traits which were influenced by different photoperiods (Zhang K. et al., 2017). Doust et al. (2017) examined flowering responses in short (8 or 12 h light) and long (16 h light) photoperiods in a QTL mapping population, and demonstrated differences between short and long day regulation, with the 8 and 12 h photoperiods sharing QTL on chromosomes 4 and 5, and the 12 and 16 h having a shared QTL on chromosome 8. In addition there were QTLs on chromosomes 2 and 3 that were unique to the 16 h photoperiod. Mauro-Herrera et al. (2013) identified 16 flowering time QTLs across eight independent trials with varying climatic and photoperiod conditions, with domesticated foxtail millet alleles contributing to increased days to flowering. These QTL regions contained many candidate flowering genes identified from rice, maize, sorghum, and Arabidopsis, suggesting that the major genetic components in Setaria flowering and photoperiod response pathways are the same as in other plant species, even if the regulatory mechanisms are different between long and short day environments.

## Perspectives for Future Breeding

Foxtail millet is well adapted to harsh environments, particularly under drought (Tang et al., 2017). It serves as an important staple grain in arid and semi-arid regions of Asia, particularly in northern China and India (Diao, 2017). The modern breeding programs of foxtail millet were initiated in the 1930s in India and 1950s in China, and were mostly focused on yield-related traits, because compared with other major crops, such as rice, wheat, and maize, the yield of foxtail millet is still relatively low (Diao and Jia, 2017a). However, global climate change, coupled with increasing population and reduction in arable lands, is adversely affecting cereal grain production in multiple regions (Wheeler and von Braun, 2013; Asseng et al., 2014). Stress tolerant foxtail millet is a potential crop that would be suitable for growing in drier and warmer conditions with few available inputs, particularly in developing countries in Asia and Africa (Huang et al., 2016).

There have been several studies exploring drought stress resistance traits in Setaria. Qie et al. (2014) investigated drought tolerance-related QTLs controlling germination and early seedling growth, using an F7 population of a cross between foxtail millet *Yugu1* and green foxtail W53, and identified a total of 18 QTLs. Tang et al. (2017) compared the transcriptomes of drought-tolerant foxtail millet *Yugu1* and drought-sensitive *An04*, and suggested that there was intense transcriptomic remodeling caused by genotype × drought stress interactions. Qi et al. (2013) studied differential gene expression patterns under PEG-induced drought treatment and found a total of 2,824 drought-responsive genes, including a large number of small RNAs and long non-coding RNAs that were actively involved in regulating drought-responsive genes. A dehydration-responsive element binding protein gene, *SiDREB2* (Lata et al., 2011; Lata and Prasad, 2014) and an ABA-responsive DREB, *SiARDP* (Li et al., 2014), were found to contribute to drought tolerance in foxtail millet. Peng et al. (2010) reported that the overexpression of phospholipase D (PLD) from foxtail millet could enhance the drought tolerance in Arabidopsis by elevating the sensitivity to ABA. These studies suggest that there is much scope for future breeding of foxtail millet with enhanced drought tolerance that may provide new opportunities for increased food production in marginal crop production areas. Besides stress tolerance, other traits like resistance to leaf rust or blast diseases, and pests such as nematodes and other insects, are also important future breeding goals.

The traditional “phenotype-to-gene” forward genetics approach has been applied in the breeding and basic research of Setaria for many decades. Although several QTLs have been successfully identified from agronomically important traits, as reviewed above, the fine mapping of candidate genes is still challenging, mostly due to the lack of high density marker maps and high-quality reference genomes (Huang et al., 2016). However, the application of next generation sequencing in diverse collections of foxtail millet cultivars (Jia et al., 2013b) and wild green foxtail accessions (Huang and Feldman, 2017) are enabling the discovery of genomic variations on a large-scale. Meanwhile, the use of bulked segregant analysis (BSA) to quickly analyze mutant collections generated by chemical mutagenesis (Jiang et al., 2017) is accelerating Setaria gene discovery (Xue et al., 2016; Fan et al., 2017; Huang et al., 2017). New genes contributing to leaf color (Li et al., 2016), height, and inflorescence architecture (Masumoto et al., 2016; Huang et al., 2017; Xiang et al., 2017; Yang et al., 2018) have been successfully identified with this approach, substantially advancing our understanding of important traits.

In addition to forward genetics, transgenic-based reverse genetics tools are also being developed. Green foxtail can be easily transformed, unlike other panicoid model systems such as maize, sorghum, and Brachypodium, which are recalcitrant to transformation. Agrobacterium-mediated transformation methods starting with mature seeds have been successfully established (Van Eck et al., 2011, 2017; Van Eck and Swartwood, 2015), and faster floral-dip transformation methods have also been proposed (Martins et al., 2015; Saha and Blumwald, 2016, 2017). The rise of genome editing via CRISPR/Cas9 system will also enhance the future of Setaria genetics (Kikuchi et al., 2017; Zhu et al., 2017). The development of the Setaria genetic toolkit and resources will accelerate gene discovery and functional genomics analysis, benefiting not only millet breeding programs, but also enabling translational research from Setaria to other panicoid crops.

## Conclusion

The Setaria system provides a window into domestication processes in the panicoid grasses that expands the discoveries that have already been made in the major crops of sorghum and maize. Differences between foxtail millet and those crops include an experimentally amenable wild ancestor that is widespread in temperate latitudes of the world and a domestication process that occurred at temperate latitudes, meaning that selection during improvement for adaptation to temperate latitudes was likely less intense than for maize and sorghum. The evidence from the traits presented here is that some conservation of genetic programs for domestication is shared between Setaria and other grasses, although definitive studies are still lacking. However, the Setaria system is not yet fully developed, and there are several areas that could be improved for future studies. These include a great need for additional and larger mapping populations, especially multi-parental mapping populations that will increase the power to identify causal genes. This approach has been very powerful for the sorghum, maize, rice, and Arabidopsis communities (Buckler et al., 2009; Kover et al., 2009; McMullen et al., 2009; Bandillo et al., 2013; Holland, 2015; Ongom and Ejeta, 2017), and would be of undoubted value for Setaria. Availability of genetic resources is also an issue, as the great diversity within China is essentially unavailable to outside researchers. Therefore, studies on the full diversity of the crop, foxtail millet, are limited to Chinese researchers at this time. Fortunately, studies on North American green foxtail wild diversity reveal that much of the species diversity is found there (Huang et al., 2014; Huang and Feldman, 2017; Schroder et al., 2017), a finding that will be important for future researchers. Another important aspect of the Setaria system is the promise of easy transformability, although there is as yet no published reports of transformation being used to confirm gene function. Routine transformation methods, including the possibility of a successful floral dip protocol, will help propel Setaria into a powerful system for gene discovery, as well as illuminating its domestication history and enhancing its future potential as a crop for a changing world.

## Author Contributions

AD conceived the idea. HH and MM-H compiled genetic studies. HH, MM-H, and AD interpreted the data, wrote, and revised the manuscript.

## Conflict of Interest Statement

The authors declare that the research was conducted in the absence of any commercial or financial relationships that could be construed as a potential conflict of interest.

## Footnotes

1 http://phytozome.jgi.doe.gov/
